# Bacteriocins as Alternative Antimicrobial Agents for Wound-Associated Infections: Mechanisms, Activity Against Biofilms and Translational Potential

**DOI:** 10.3390/antibiotics15090884

**Published:** 2026-09-09

**Authors:** Magdalena Szemraj, Monika Sienkiewicz

**Affiliations:** Department of Pharmaceutical Microbiology and Microbiological Diagnostics, Faculty of Pharmacy, Medical University of Lodz, Muszynskiego 1, 90-151 Łódź, Poland

**Keywords:** bacteriocins, antimicrobial peptides, biofilms, antibiofilm activity, antimicrobial resistance, wound pathogens, drug delivery systems, therapeutic applications

## Abstract

**Background/Objectives**: Skin and soft tissue infections (SSTIs) remain a major clinical challenge due to the increasing prevalence of antimicrobial resistance and biofilm-associated pathogens. Conventional antibiotics are often limited by reduced efficacy, recurrent infections, and disruption of the resident skin microbiota. Consequently, bacteriocins have emerged as promising alternative or adjunctive antimicrobial agents for the treatment of skin and wound infections. **Methods**: A literature review was conducted using the PubMed, Scopus, and Web of Science databases. Experimental in vitro, ex vivo, and in vivo studies investigating bacteriocins in skin and wound infection models were analyzed, with a focus on antimicrobial activity, antibiofilm efficacy, activity against antimicrobial-resistant pathogens, and formulation strategies designed to improve therapeutic performance. **Results**: Available evidence demonstrates that numerous bacteriocins exhibit potent antimicrobial activity against clinically relevant skin-associated pathogens, particularly *Staphylococcus aureus*, including methicillin-resistant strains (MRSA). Several bacteriocins also showed significant antibiofilm properties and synergistic interactions with conventional antibiotics, resulting in enhanced bacterial eradication and reduced risk of resistance development. Experimental infection models further support their therapeutic potential in wound-associated infections. Additionally, advanced delivery platforms, including hydrogels, wound dressings, nanofibers, and lipid-based nanoparticles, improved peptide stability, sustained release, and local antimicrobial efficacy. **Conclusions**: Bacteriocins are promising candidates for the prevention and treatment of skin and wound infections due to their antimicrobial and antibiofilm activity, low propensity for resistance development, and suitability for topical administration. However, further studies addressing formulation optimization, safety, pharmacokinetics, and clinical validation are required before bacteriocin-based therapies can enter routine clinical practice.

## 1. Introduction

Skin and soft tissue infections (SSTIs) are common bacterial diseases occurring in both community and healthcare settings [1]. They range from superficial conditions, such as impetigo and folliculitis, to more severe and invasive infections, including cellulitis, abscesses, surgical site infections, and infected acute or chronic wounds [2,3]. The clinical burden of SSTIs is greatest among individuals with diabetes, immunosuppression, burns, and chronic wounds, in whom dysbiosis, biofilm formation, and impaired tissue repair synergistically contribute to persistent infection and delayed healing [1,4]. Gram-positive bacteria such as *Staphylococcus aureus* and *Streptococcus pyogenes* are predominant etiological agents of skin infections. Regarding chronic wounds, Gram-negative microorganisms are more frequently observed and they include *Pseudomonas aeruginosa* and representatives of Enterobacterales, which further complicates the treatment and promotes disease relapses [5,6]. In recent years, increasing prevalence of antibiotic-resistant strains, such as methicillin-resistant *S. aureus* (MRSA), have significantly limited therapeutic options and complicated the management of skin infections. The global rise of antimicrobial resistance (AMR) represents a major public health concern and indicates an urgent need for alternative or complementary antimicrobial strategies. Conventional antibiotics used in treatment of skin infections often exhibit broad-spectrum activity, which may disrupt the native skin microbiota and contribute to resistance development [1,7]. Moreover, the efficacy of antibiotics is frequently reduced in biofilm-associated infections, which are commonly observed in chronic wounds [6]. These issues aroused interest in novel antimicrobial agents with distinct mechanisms of action and a lower tendency to induce resistance. Bacteriocins are ribosomally synthesized antimicrobial peptides produced by bacteria as part of their competitive survival strategies. They show strong antibacterial activity, often with high specificity toward closely related or clinically relevant pathogens [8,9]. Several features make bacteriocins particularly attractive candidates for the treatment of skin infections. These peptides often display rapid bactericidal activity at low concentrations, effectiveness against antibiotic-resistant pathogens, and a lower propensity for resistance development compared with many conventional antibiotics [8,10]. Importantly, bacteriocins can be applied locally, which is a major advantage in dermatological and wound-related applications, as topical administration minimizes systemic exposure and potential toxicity [11]. Furthermore, some bacteriocins demonstrated activity against biofilm-forming bacteria, suggesting their potential utility in chronic wound management [12,13,14].

**Figure 1:**
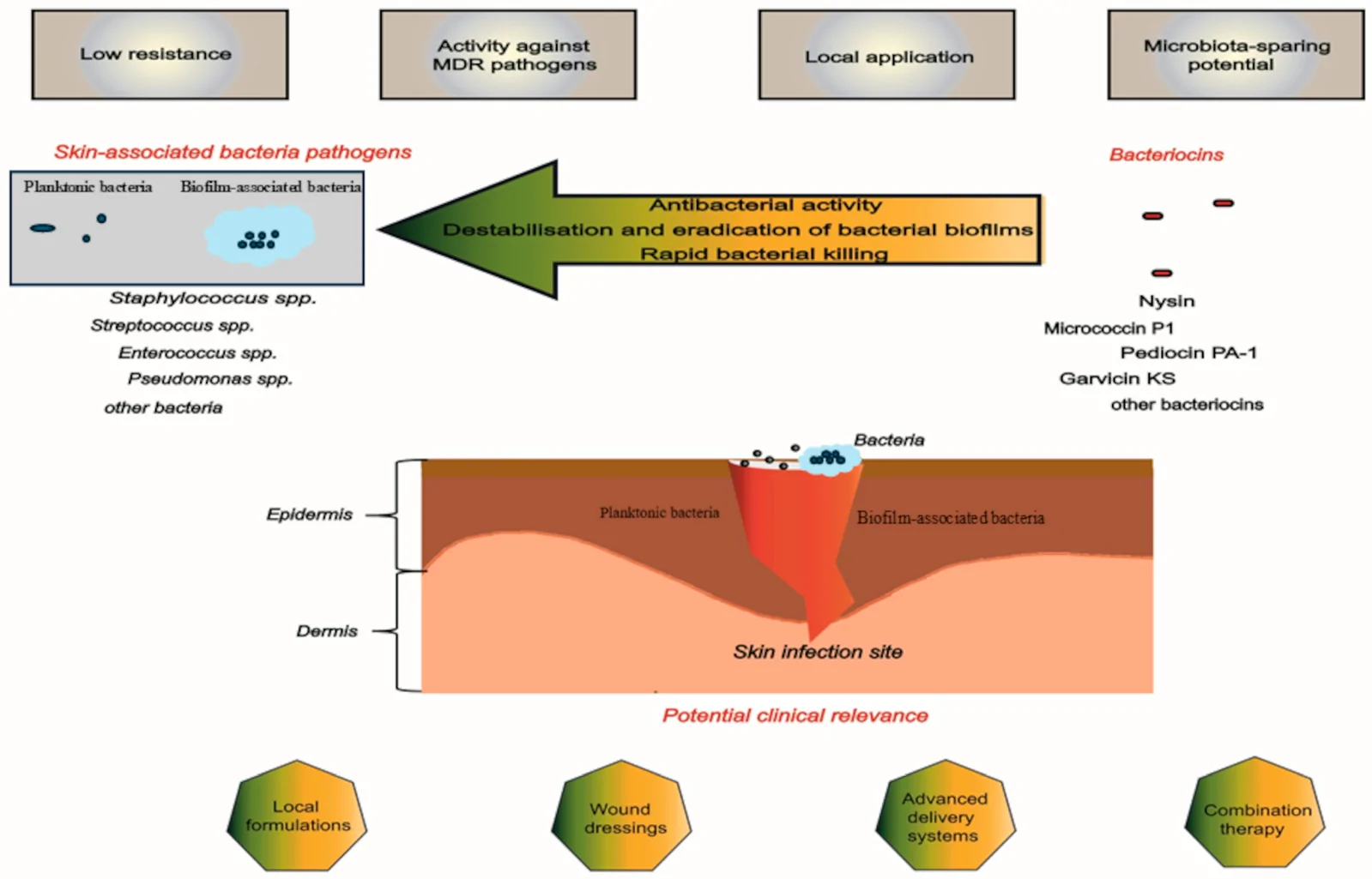
Therapeutic Potential of Bacteriocins in Skin Infections.

Despite a growing body of evidence supporting antimicrobial properties of bacteriocins, their therapeutic application in skin infections is still at an early stage of research. Most studies carried out so far have focused on in vitro antibacterial activity and there have been fewer investigations on in vivo efficacy conducted with the use of relevant skin or wound infection models. Furthermore, challenges related to peptide stability, delivery, immunogenicity, and regulatory approval continue to limit clinical translation of bacteriocin-based therapeutics. Recent advances in formulation strategies, such as encapsulation, hydrogel-based delivery systems, and combination therapies with conventional antibiotics, offer promising solutions to overcome these limitations. Figure 1 shows therapeutic applications of bacteriocins in skin and wound infections.

The figure demonstrates bacteriocin activity at the skin surface and within wounded tissue, targeting planktonic bacteria and biofilm-associated pathogens commonly involved in skin infections. Major skin-associated pathogens, including *Staphylococcus* spp., *Streptococcus* spp., *Enterococcus* spp., *Pseudomonas* spp., are shown. Therapeutic applications of bacteriocins are described, including topical formulations, wound dressings, advanced delivery systems, and combination therapy with conventional antibiotics. Key advantages supporting clinical translation, such as activity against multidrug-resistant pathogens and suitability for localized treatment, are highlighted.

The aim of this review is to critically evaluate the current evidence regarding the use of bacteriocins as alternative antimicrobial agents for the treatment of skin and wound infections. We summarize data from in vitro, ex vivo, and in vivo studies investigating the efficacy of bacteriocins against clinically relevant skin-associated pathogens, discuss their mechanisms of action and therapeutic potential, and highlight current limitations and future perspectives for the clinical translation of bacteriocin-based therapies. While previous reviews have primarily focused on bacteriocin biology, classification, mechanisms of action, or their general applications against antimicrobial-resistant pathogens, the present review specifically integrates evidence related to skin- and wound-associated infections. Particular attention is given to antibiofilm activity, experimental infection models, topical delivery systems, and translational challenges that may influence future clinical implementation.

## 2. Materials and Methods

A literature narrative review was conducted using the PubMed, Scopus, and Web of Science databases to identify publications addressing bacteriocins and their potential applications as antimicrobial agents in skin and wound infections. Literature searches included articles published up to December 2025. The search strategy was based on combinations of the following keywords: bacteriocins, antimicrobial peptides, biofilms, antibiofilm activity, antimicrobial resistance, wound pathogens, wound infections, drug delivery systems, therapeutic applications, and wound healing. Representative search strings included: (“bacteriocins” AND “wound infections”), (“bacteriocins” AND “biofilm”), (“bacteriocins” AND “skin infections”), (“bacteriocins” AND “drug delivery systems”), and (“bacteriocins” AND “antimicrobial resistance”). Additional relevant publications were identified through manual screening of the reference lists of selected articles, including those made in 2026. Titles and abstracts of the identified records were screened for relevance to the scope of the review. Potentially eligible publications were subsequently assessed in full text. Priority was given to peer-reviewed original research articles reporting experimental data from in vitro, ex vivo, and in vivo studies. Review articles were included selectively to provide background information, classification frameworks, mechanistic insights, and broader context regarding bacteriocin biology, antimicrobial resistance, skin infections, and biofilm-associated diseases. Conference abstracts, non-peer-reviewed publications, duplicate records, and studies lacking sufficient methodological information were excluded from further analysis.

The selected literature was evaluated regarding: (i) bacteriocin classification and mechanisms of action, (ii) antimicrobial activity against clinically relevant skin- and wound-associated pathogens, (iii) antibiofilm properties, (iv) activity against antimicrobial-resistant microorganisms, (v) evidence obtained from experimental skin and wound infection models, and (vi) translational aspects, including formulation strategies and delivery systems designed to improve bacteriocin stability, bioavailability, and therapeutic efficacy.

## 3. Bacteriocins: Definition, Classification, and Mechanisms of Action

Bacteriocins are a heterogeneous group of ribosomally synthesized antimicrobial pep-tides and proteins produced by bacteria and, less frequently, archaea, which exert bactericidal or bacteriostatic activity primarily against phylogenetically related microorganisms [8,15]. Unlike most conventional antibiotics, which are non-ribosomally synthesized secondary metabolites, bacteriocins are ribosomally synthesized antimicrobial peptides or proteins encoded by specific structural genes and often subjected to post-translational modifications. They frequently display a relatively narrow spectrum of activity directed against closely related microorganisms [16]. These specific properties reflect their ecological function as molecular tools mediating microbial competition, niche establishment, and population structure within complex microbial communities, including soil, aquatic environments, fermented foods, and host-associated microbiota [15]. Due to their proteinaceous nature, bacteriocins are generally susceptible to proteolytic degradation, which contributes to their low environmental persistence and reduced toxicity towards eukaryotic cells, making them attractive candidates for biomedical and biotechnological applications [17].

**Figure 2:**
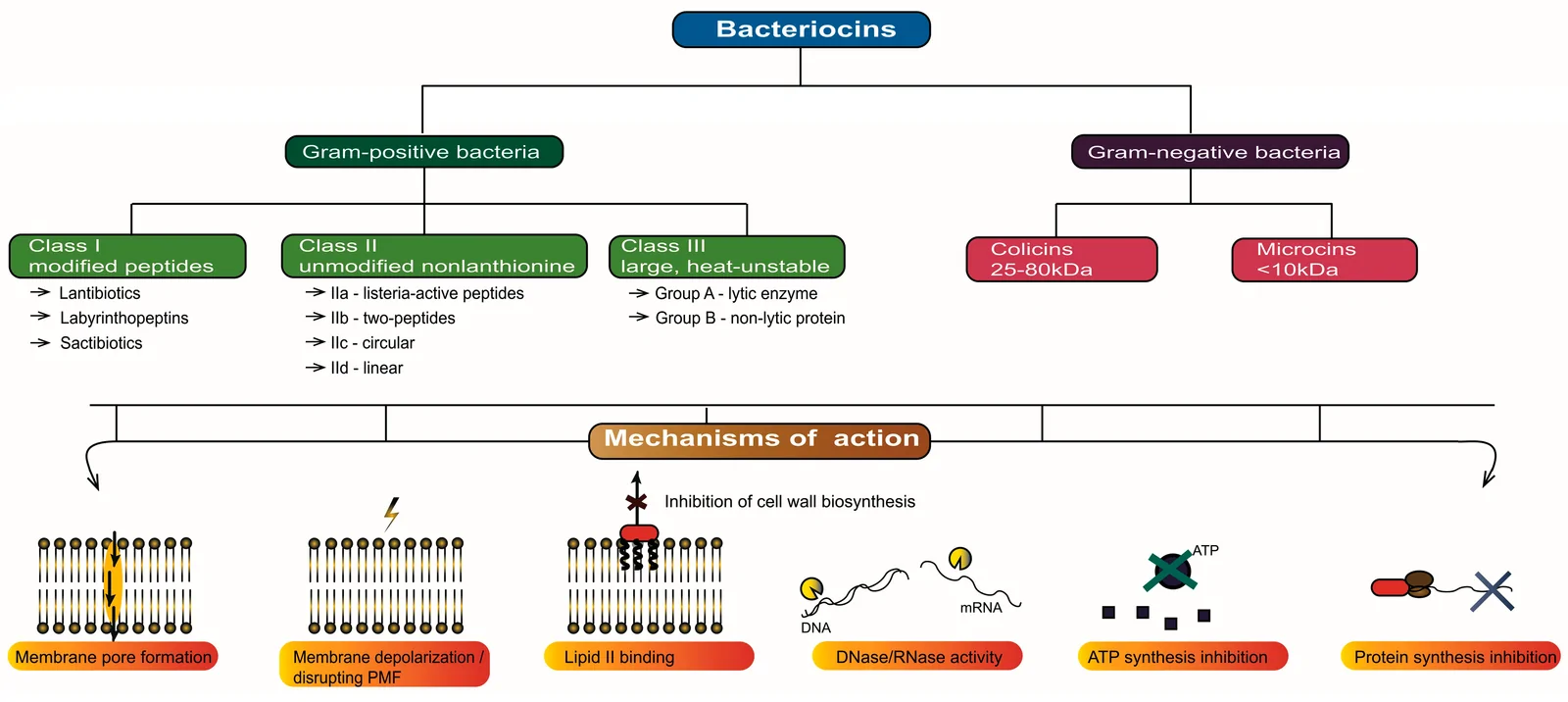
Classification of bacteriocins and overview of their possible mechanisms of action.

Bacteriocins belong to ribosomally synthesized peptides that can undergo post-translational modifications [18]. Their genetic determinants are typically organized in operons comprising structural genes, modification enzymes, dedicated transporters, immunity proteins, and regulatory elements. They all together ensure efficient biosynthesis, secretion, and self-protection of the producing strain [9]. This modular genetic architecture facilitates rapid evolutionary diversification and horizontal gene transfer, contributing to remarkable structural and functional diversity of bacteriocins [8]. On the basis of molecular mass, post-translational modifications, heat stability, and mechanism of action, bacteriocins are traditionally classified into several major groups [8,9,16]. The main classification of bacteriocins based on the cell wall type of producing organisms is shown in Figure 2.

The figure presents the main classes of bacteriocins produced by Gram-positive and Gram-negative bacteria, categorized according to their size, structure, and post-translational modifications. Importantly, the mechanisms of action illustrated in the scheme do not correspond to specific bacteriocin classes but represent an overview of different antimicrobial strategies employed by bacteriocins. In general, lantibiotics (Class I) most frequently act via Lipid II binding and membrane pore formation, Class II bacteriocins predominantly exert activity through membrane permeabilization and depolarization, whereas large bacteriocins (Class III) often display enzymatic bacteriolytic activity. In contrast, colicins and microcins, produced by Gram-negative bacteria, typically target intracellular processes, including nucleic acid degradation, inhibition of protein synthesis, or interference with cellular energy metabolism. Individual bacteriocins can act through one or multiple mechanisms, depending on their structure and target organism.

Bacteriocins of Gram-positive bacteria refer to three main classes. Class I bacteriocins, commonly referred to as lantibiotics, are small peptides, typically below 5 kDa, that undergo extensive post-translational modifications, including dehydration of serine and threonine residues, followed by intramolecular cyclization with cysteine residues to form lanthionine and methyllanthionine bridges [9,19]. These thioether cross-links generate rigid, polycyclic structures that are essential for antimicrobial activity and resistance to proteolysis. Well-characterized lantibiotics such as nisin, subtilin, and epidermin trigger their activity primarily through high-affinity binding to lipid II, a universally conserved precursor in bacterial peptidoglycan biosynthesis [20]. This interaction not only sequesters lipid II and blocks cell wall synthesis but also facilitates formation of stable membrane pores, which results in rapid depolarization, leakage of intracellular ions, and cell death [8,20,21]. The dual targeting of lipid II and the cytoplasmic membrane is thought to contribute to the high potency of lantibiotics and their relatively low propensity for resistance development. Class II bacteriocins comprise a large and diverse group of small, heat-stable peptides, generally below 10 kDa, that lack extensive post-translational modifications [22]. This class includes pediocin-like bacteriocins (class IIa), characterized with strong anti-listerial activity, two-peptide bacteriocins (class IIb), whose antimicrobial function depends on the synergistic interaction of two distinct peptides, peptides with a circular structure (class IIc) as well as linear peptides which do not exhibit activity against Listeria (class IId) [8,23]. Many class II bacteriocins act by electrostatic attraction to negatively charged components of the bacterial cytoplasmic membrane, followed by insertion into the lipid bilayer and formation of pores that dissipate the proton motive force [21]. Importantly, several class II bacteriocins rely on specific membrane-associated receptors, most notably the mannose phosphotransferase system (Man-PTS), which serves as a docking site and determines species-specific sensitivity [24]. Alterations in these receptor components can result in bacteriocin resistance, highlighting the receptor-dependent nature of their antimicrobial activity. Class III bacteriocins, also known as bacteriolysins, are high-molecular-weight, heat-labile proteins, exceeding 30 kDa, that differ fundamentally from classes I and II in both their structure and mechanism of action [16]. These bacteriocins do not form pores in the cytoplasmic membrane, but they typically exhibit enzymatic activity directed against structural components of the bacterial cell envelope, most commonly peptidoglycan. By catalyzing the hydrolysis of specific bonds within the cell wall, bacteriolysins induce osmotic lysis of susceptible cells [8]. Bacteriocins produced by Gram-negative bacteria are structurally and functionally diverse and are traditionally classified based on their molecular weight, structure, mode of action, and secretion mechanisms [11,25]. The two principal groups are colicins and microcins, with an additional category comprising phage tail-like bacteriocins [11,26]. Colicins are high-molecular-weight protein bacteriocins (typically 25–80 kDa) produced predominantly by *E. coli* and closely related members of the *Enterobacteriaceae* family [25,27]. They are generally plasmid-encoded and released by the producer during cell lysis. They also exhibit narrow-spectrum activity directed mainly against closely related Gram-negative bacteria [27,28]. Based on their mechanisms of action, colicins are commonly subdivided into three functional groups. Pore-forming colicins disrupt the integrity of the cytoplasmic membrane by forming voltage-dependent channels; nuclease colicins possess DNase, RNase, or tRNase activity leading to degradation of nucleic acids; finally, colicins inhibiting cell wall synthesis interfere with peptidoglycan biosynthesis [11,28,29]. Colicin activity requires specific outer membrane receptors and translocation systems, such as the Tol or Ton systems, which contribute to their narrow target [27,28]. Microcins are low-molecular-weight bacteriocins (<10 kDa), including very small peptides (<5 kDa). They undergo extensive post-translational modifications and can form slightly larger peptides (5–10 kDa) that are unmodified or minimally modified [11,29]. Microcins exhibit antimicrobial activity through diverse mechanisms, including membrane depolarization, inhibition of DNA or protein synthesis, and disruption of essential metabolic pathways [30]. A distinct group of bacteriocins produced by Gram-negative bacteria comprise phage tail-like bacteriocins, also referred to as tailocins [25,31]. These high-molecular-weight complexes structurally resemble bacteriophage tails but they lack genetic material and are produced by various Gram-negative species, including *P. aeruginosa*, where they are known as R-type (contractile) and F-type (flexible) pyocins [11,31]. Tailocins show considerable bactericidal activity by binding to specific surface receptors on target cells, followed by rapid membrane depolarization or cell envelope disruption, resulting in cell death [26,31].

Overall, the mechanistic diversity of bacteriocins reflects their pronounced structural heterogeneity and evolutionary adaptation to specific ecological pressures. By targeting essential bacterial components, such as cell membranes, lipid II, nucleic acids, or cell wall structures, often with high specificity toward particular species or strains, bacteriocins exert potent antimicrobial activity while minimizing off-target effects on non-target microorganisms [8,15]. This selective mode of action also influences the nature and dynamics of resistance development. In comparison to conventional antibiotics, resistance to bacteriocins appears to emerge less frequently and to develop more slowly. However, this phenomenon remains an active area for research [17]. Consequently, bacteriocins are increasingly regarded as promising alternatives or complementary agents to traditional antimicrobials in fields such as food preservation and veterinary medicine, and potentially in human therapy, particularly in the context of the global rise in antimicrobial resistance [16].

## 4. Skin Structure and Bacterial Skin Infections

The skin constitutes the largest organ of the human body and functions as a highly specialized physical, chemical, and immunological barrier protecting the host from environmental insults and microbial invasion. Structurally, it is composed of the epidermis, dermis, and hypodermis, which together provide mechanical resilience, immune surveillance, and tissue homeostasis [32,33]. The outermost layer of the epidermis, the stratum corneum, serves as a barrier and consists of terminally differentiated keratinocytes, embedded in a lipid matrix rich in ceramides, cholesterol, and free fatty acids [34]. This structure limits transepidermal water loss, maintains an acidic pH on the skin surface, and, together with antimicrobial lipids and the resident microbiota, contributes to a protective environment against pathogenic microorganisms [34,35]. In addition to its physical properties, the skin is an active immune organ equipped with a complex network of innate and adaptive defense mechanisms. Keratinocytes express pattern-recognition receptors (PRRs), including Toll-like receptors and NOD-like receptors, enabling rapid detection of microbial components and induction of inflammatory and antimicrobial responses [36,37]. Among Toll-like surface receptors are TLR2 (Toll-like receptor 2) recognizing peptidoglycan of Gram-positive bacteria and TLR4 (Toll-like receptor 4) recognizing LPS of Gram-negative. NOD-like receptors detect pathogens inside host cells in cytosol. In response to microbial stimulation, keratinocytes and other skin-resident cells produce antimicrobial peptides such as β-defensins, cathelicidin LL-37, and dermcidin. Apart from exerting direct antimicrobial activity against bacteria, fungi, and certain viruses, these peptides also exhibit immunomodulatory properties, including chemotactic activity and regulation of cytokine production [37,38]. These mechanisms operate together with immune cells, including Langerhans cells, dermal dendritic cells, macrophages, and T lymphocytes, in maintenance of cutaneous immune homeostasis [39]. The skin is colonized by a diverse microbiota that plays a fundamental role in maintaining barrier integrity and preventing pathogen overgrowth. Commensal microorganisms such as *Staphylococcus epidermidis*, *Cutibacterium acnes*, and *Corynebacterium* spp. contribute to colonization resistance by competing for nutrients and adhesion sites, producing antimicrobial molecules, including bacteriocins and other antimicrobial peptides, and modulating host immune responses [40,41,42,43]. In particular, certain commensal strains can stimulate antimicrobial peptide production in keratinocytes and directly inhibit pathogens such as *S. aureus*, highlighting the importance of host–microbiota interactions in cutaneous defense [40,42]. Disruption of the skin barrier or dysbiosis of the resident microbiota may compromise these protective mechanisms and predispose individuals to cutaneous infections [33,34]. Bacterial skin infections develop when microorganisms overcome physical and immunological defensive properties of the skin, often facilitated by microtrauma, chronic inflammation, excessive moisture, or systemic conditions such as diabetes, vascular disease, or immunosuppression [44]. Gram-positive bacteria, particularly *S. aureus* and *S. pyogenes*, remain predominant etiological agents of skin and soft tissue infections. However, Gram-negative and also opportunistic pathogens are increasingly encountered in healthcare-associated infections, chronic wounds, and immunocompromised patients [2,45,46].

*S. aureus* is a predominant etiological agent of both superficial and invasive skin and soft tissue infections and represents the most frequently isolated pathogen in SSTIs worldwide [47]. Its success as a cutaneous pathogen is attributed to a complex repertoire of virulence determinants that facilitate colonization, immune evasion, and tissue destruction. These determinants include microbial surface components recognizing adhesive matrix molecules (MSCRAMMs) such as clumping factors and fibronectin-binding proteins, as well as secreted cytolytic toxins (e.g., α-toxin, Panton–Valentine leukocidin, and TSST-1) and multiple immune-modulating proteins [48]. In addition, *S. aureus* readily forms structured biofilms on damaged skin and wound surfaces, which markedly increases tolerance to antimicrobial therapy and protects bacteria from host immune clearance [6,49]. Methicillin-resistant *S. aureus* strains are of particular clinical concern because they combine extensive antimicrobial resistance with enhanced persistence in chronic wounds and healthcare environments [45,48]. Horizontal gene transfer, biofilm-associated growth, and colonization reservoirs in the skin and nares contribute to recurrent infections and therapeutic failure. *S. pyogenes* (group A streptococcus, GAS) similarly causes a wide spectrum of cutaneous diseases, ranging from impetigo and erysipelas to invasive infections such as necrotizing fasciitis and streptococcal toxic shock syndrome [50]. Its pathogenicity relies on multiple coordinated virulence factors. The surface M protein, encoded by the emm gene, acts as a major anti-phagocytic determinant by interfering with complement deposition and opsonization [51,52]. In addition, GAS secretes a broad array of enzymes and toxins, including streptolysins, DNases, streptokinase, and superantigenic exotoxins such as streptococcal pyrogenic exotoxins, which promote tissue invasion, immune dysregulation, and systemic inflammation [52,53]. Not only classical Gram-positive pathogens but also Gram-negative bacteria play an increasingly recognized role in skin and wound infections, particularly in chronic wounds, burns, and healthcare-associated settings [1,5,43]. *P. aeruginosa* is one of the most clinically relevant opportunistic pathogens in this context and is frequently isolated from chronic ulcers and burn wounds, where it colonizes areas of disrupted skin barrier and moist microenvironments [54]. The pathogenicity of *P. aeruginosa* relies on a multifactorial virulence strategy that includes *quorum-sensing*-regulated production of exotoxin A, elastases, pyocyanin, and rhamnolipids, as well as secretion systems and extracellular matrix-degrading enzymes that impair tissue repair and host defense responses [55,56]. A critical feature of this organism is the ability to form structured biofilms on wound surfaces; the biofilm matrix, enriched in alginate and other polysaccharides, protects bacteria from antimicrobial agents and immune clearance and contributes to delayed wound healing and persistent infection [57,58]. *Acinetobacter baumannii* has also emerged as an important opportunistic pathogen in traumatic, surgical, and combat-related wounds, particularly in hospitalized and immunocompromised patients [59]. It is considered a member of the ESKAPE group of multidrug-resistant pathogens and is strongly associated with healthcare-associated infections [60]. This species readily forms biofilms on biotic and abiotic surfaces and exhibits extensive antimicrobial resistance, which significantly complicates therapy and promotes persistent colonization of wounds and medical devices [61,62]. Members of the Enterobacterales, including *E. coli*, *Klebsiella pneumoniae*, and *Proteus* spp., are frequently isolated from chronic wounds, diabetic foot ulcers, and surgical site infections, where they often coexist with Gram-positive cocci and non-fermenting Gram-negative rods [63,64]. These organisms readily exploit compromised skin integrity, ischemia, and inflammatory microenvironments, contributing to polymicrobial infections associated with increased tissue damage and delayed healing. Their pathogenicity is supported by the production of endotoxin (lipopolysaccharide), proteases, hemolysins, and siderophores, as well as by their capacity to participate in mixed-species biofilms that enhance tolerance to antibiotics and host immune defenses [65,66]. At the molecular level, bacterial skin infections are associated with disruption of the epidermal barrier, including alterations in stratum corneum lipid organization and associated proteins, which impair barrier integrity and facilitate microbial adhesion and invasion [33,34]. Pathogens such as *S. aureus* can directly perturb lipid composition within the stratum corneum, exacerbating barrier dysfunction and local inflammation [67]. Additionally, dysregulated expression of endogenous antimicrobial peptides, including β-defensins, cathelicidin LL-37, and dermcidin, together with aberrant inflammatory signaling, can further impair barrier integrity and host immune defenses, thereby increasing susceptibility to cutaneous infections and chronic inflammation [37,41,68]. Biofilm formation on the skin surface and within wound beds represents a central factor in the chronicity of many infections, as biofilm communities display markedly increased tolerance to antimicrobial agents and resistance to phagocytosis and oxidative killing [43,64]. Moreover, interspecies interactions within polymicrobial biofilms, including metabolic cooperation and quorum-sensing cross-communication, can enhance overall pathogenicity and further complicate treatment [63].

Summing up, these findings indicate that bacterial skin infections are multifactorial processes driven by the interplay between skin barrier integrity, immune responses, microbiota composition, and pathogen-specific virulence traits. The growing awareness of the role of opportunistic bacteria in skin infections and limited efficacy of antibiotics against biofilm-associated bacteria require implementation of alternative or adjunctive antimicrobial strategies. In this context, bacteriocins, together with other antimicrobial peptides, have become the focus of attention as targeted agents with narrow or selective spectra of activity.

## 5. Antimicrobial Activity of Bacteriocins Against Bacterial Species Relevant to Skin and Wound Infections

The studies summarized in Table 1 focus on original experimental works evaluating bacteriocin activity against clinically relevant pathogens commonly associated with skin and wound infections under planktonic, biofilm, and infection-mimicking conditions. Particular emphasis is placed on antibiofilm efficacy and activity against multidrug-resistant isolates, as these parameters are critical determinants of therapeutic relevance in chronic wound management. The table includes different bacteriocins for which the minimum concentration inhibiting bacterial growth was determined. These data provide a structured overview of the current preclinical evidence supporting bacteriocins as targeted antimicrobial agents in dermatology and wound care.

The studies summarized in Table 1 demonstrate that bacteriocins consistently exhibit strong activity against Gram-positive wound-associated pathogens, particularly *Staphylococcus* spp. However, direct comparison of antimicrobial efficacy across studies is complicated by substantial methodological heterogeneity. Differences include the use of reference versus clinical multidrug-resistant isolates, evaluation of planktonic cells versus biofilms, variability in bacteriocin purification methods, and the application of different antimicrobial endpoints, including MIC, MBC, and biofilm inhibition assays. Furthermore, activity against Gram-negative pathogens appears less consistent than against Gram-positive bacteria, largely reflecting biological differences in cell envelope structure rather than inherent limitations of individual bacteriocins.

Bacteriocins of Gram-positive origin, particularly lantibiotics and related peptides such as nisin, lacticin 3147, gallidermin, epidermicin NI01 and enterocin AS-48, primarily act by binding to lipid II or by inducing rapid membrane permeabilization, leading to depolarization and cell death in susceptible bacteria [71,73,78,79]. Because Gram-positive pathogens lack an outer membrane, these peptides can directly access cytoplasmic membrane targets, which explains their consistent and strong activity against *S. aureus*, including MRSA, one of dominant pathogens of skin and soft-tissue infections. Among the analyzed compounds, nisin A remains the most extensively characterized bacteriocin, with reproducible MIC values typically in the range of 2–16 µg/mL across independent studies [13,14,69]. These findings are in agreement with its well-established mechanism of action, which involves binding to lipid II and subsequent pore formation in the cytoplasmic membrane, leading to rapid cell death. Notably, despite relatively consistent MIC values, variability in susceptibility among *S. aureus* strains was observed, with higher MIC values reported for some clinical isolates, particularly MRSA [13]. This suggests that intrinsic or adaptive resistance mechanisms, including alterations in membrane composition or surface charge, may influence bacteriocin efficacy. An important finding across several studies is the ability of bacteriocins to inhibit biofilm formation. Nisin demonstrated antibiofilm activity at concentrations comparable to MIC values (MBIC ≈ 4 µg/mL), although eradication of mature biofilms required substantially higher concentrations (up to 256 µg/mL) [13]. This discrepancy reflects well-known increased tolerance of biofilm-associated cells. Other bacteriocins, such as enterocin AS-48, also exhibited biofilm-reducing activity [71], suggesting that membrane-active peptides may be particularly effective against early stages of biofilm formation. The reduced susceptibility of mature biofilms cannot be explained solely by increased bacterial density. Biofilm-associated microorganisms are embedded within a highly structured extracellular polymeric substance (EPS) matrix composed of polysaccharides, proteins, extracellular DNA, and host-derived components. This matrix acts as a physical and chemical barrier that can delay or reduce antimicrobial penetration, while simultaneously creating spatial gradients of nutrients, oxygen, pH, and metabolic activity within the biofilm. Consequently, bacteria located in deeper biofilm layers may be exposed to subinhibitory concentrations of antimicrobial agents, facilitating survival despite treatment [6]. In addition to limited antimicrobial penetration, biofilm-associated tolerance is strongly influenced by physiological heterogeneity. Mature biofilms contain subpopulations of metabolically inactive or slow-growing persister cells that are highly tolerant to antimicrobial exposure. These cells are not genetically resistant; however, their dormant physiological state markedly reduces susceptibility to agents targeting active cellular processes. Following treatment cessation, persister populations may repopulate the biofilm and contribute to infection recurrence, representing one of the major obstacles to successful eradication of chronic biofilm-associated infections [85]. Another important factor is quorum sensing (QS), which coordinates bacterial behavior through cell-density-dependent signaling. QS regulates biofilm maturation, virulence factor production, stress responses, and, in many species, biofilm-associated antimicrobial tolerance. The role of QS becomes particularly important in polymicrobial infections, where interspecies communication may further enhance biofilm resilience and alter susceptibility profiles. Chronic wound biofilms are rarely monospecies communities and frequently contain complex interactions between *S. aureus*, *P. aeruginosa*, Enterobacterales, and other microorganisms. Such interactions may promote cooperative survival strategies that are difficult to reproduce in simplified laboratory systems [86]. These observations suggest that inhibition of initial biofilm formation and eradication of mature biofilms should be regarded as distinct therapeutic objectives. While several bacteriocins demonstrate promising activity against early-stage biofilms, substantially higher concentrations are often required to disrupt established biofilm communities. Moreover, the predominance of monomicrobial biofilm models in the current literature limits the ability to predict bacteriocin performance under clinically relevant conditions. Future studies should therefore prioritize mature and polymicrobial biofilm systems that more accurately reflect the complexity of chronic wound infections. A limited number of studies evaluating biofilm eradication highlights a significant gap in hitherto studies.

Differences in antimicrobial potency were observed between bacteriocin classes. Highly potent bacteriocins such as epidermicin NI01 exhibited MIC values in the sub-microgram range, indicating strong antimicrobial activity even at very low concentrations [78]. Similarly, flexusin A demonstrated high potency (MIC 1 µg/mL), suggesting that *Bacillus*-derived peptides may be an underexplored source of effective antimicrobials [82]. In contrast, bacteriocins produced by lactic acid bacteria, including plantaricins, generally exhibited moderate activity (MIC 15–32 µg/mL) although certain variants showed improved efficacy [76]. This variability likely reflects differences in peptide structure, stability, and membrane interaction mechanisms. The activity of bacteriocins against Gram-negative pathogens was markedly less consistent. Although nisin demonstrated inhibitory effects against a range of Gram-negative bacteria, including *Acinetobacter* spp., *Pseudomonas* spp., and *E. coli*, significantly higher MIC values were required [70]. This observation supports the widely accepted role of the outer membrane as a barrier limiting bacteriocin penetration. Nevertheless, some bacteriocins showed promising activity against clinically relevant Gram-negative pathogens. For example, a bacteriocin from *L. plantarum* inhibited *A. baumannii* and reduced biofilm formation by nearly 60% [80], indicating potential for targeting multidrug-resistant wound pathogens. In addition, bacteriocins produced by Gram-negative bacteria, such as those from *P. aeruginosa*, displayed broader antimicrobial spectra, including activity against both Gram-positive and Gram-negative species [84]. These findings suggest that bacteriocins derived from Gram-negative organisms may have distinct mechanisms which enable them to overcome outer membrane barriers.

## 6. Experimental Evidence for the Use of Bacteriocins in Skin and Wound Infection Models

Antimicrobial properties of bacteriocins described in the preceding section demonstrate that many of these peptides and protein complexes exhibit strong activity against key skin-associated pathogens under controlled laboratory conditions. However, inhibition of planktonic bacteria alone does not allow to adequately predict therapeutic utility in dermatological infections. Skin and wound infections represent complex ecological systems in which bacteria exist within biofilms, interact with host tissues, and are exposed to proteases, inflammatory mediators, and fluctuating oxygen and nutrient availability. Consequently, translational evaluation requires experimental models that more closely reproduce the biological and physicochemical conditions present in infected skin (Table 2).

Studies summarized in Table 2 provide important evidence that the antimicrobial activity of bacteriocins observed under in vitro conditions can, to some extent, translate into efficacy in more complex infection models. These studies indicate that bacteriocins demonstrate the strongest preclinical efficacy against Gram-positive pathogens, particularly MRSA, whereas evidence supporting their activity in Gram-negative wound infections remains limited. In contrast to planktonic assays, these models incorporate key features of skin and wound infections, including biofilm formation, host–pathogen interactions, and local microenvironmental factors. Among the investigated bacteriocins, nisin A remains the most extensively studied in translational settings. In a murine skin infection model, local administration of nisin resulted in a significant reduction in MRSA burden and prevented progression of infection [91]. These findings support earlier in vitro observations and suggest that nisin retains activity in biologically relevant environments. Importantly, the therapeutic efficacy of nisin appears to be enhanced when used in combination with conventional antibiotics. A synergistic interaction between nisin and oxacillin was demonstrated using checkerboard analysis, fractional inhibitory concentration index (FICI) determination, and time-kill assays, and was subsequently confirmed in a murine skin infection model [87]. The combination significantly reduced the concentrations required to inhibit MRSA growth, enhanced bacterial clearance, and suppressed biofilm formation. Furthermore, mechanistic analyses suggested that nisin may partially restore oxacillin susceptibility by facilitating cell envelope disruption and reducing expression of the resistance-associated mecA gene [87]. This highlights the potential of bacteriocins as adjuvants capable of restoring antibiotic susceptibility in resistant pathogens. A key advantage of bacteriocins in translational models is their activity against biofilm-associated infections. For example, bacteriocin XJS01 produced by *Lactobacillus salivarius* disrupted established MDR *S. aureus* biofilms in a wound infection model and significantly reduced biofilm-associated bacterial populations [12]. Similarly, combination therapies involving bacteriocins demonstrated enhanced efficacy against biofilm-forming MRSA. Micrococcin P1, when combined with rifampicin, demonstrated synergistic activity in checkerboard-based combination susceptibility assays, resulting in substantial reductions in the MIC values of both agents compared with monotherapy. The interaction was subsequently validated in a murine MRSA wound infection model, where the combination efficiently eradicated infection, prevented relapse, and reduced the emergence of resistant variants [88]. A more complex formulation combining garvicin KS, micrococcin P1, and penicillin G contributed to complete eradication of MRSA from infected wounds and reduced the occurrence of resistance compared to antibiotic treatment alone [89]. Synergistic interactions in this study were assessed using a microplate checkerboard assay, in which serial two-fold dilutions of the antimicrobials were combined and tested for MRSA. Synergy was quantified by calculating the fractional inhibitory concentration (FIC), defined as the sum of the FIC values of the individual components. According to the authors’ criteria, FIC values ≤ 0.5 indicated synergy for two-component combinations, whereas FIC values ≤ 0.75 were considered synergistic for three-component formulations. Using this approach, the garvicin KS–micrococcin P1–penicillin G combination met predefined criteria for synergy and demonstrated significantly greater antibacterial activity than the individual antimicrobial agents alone. Importantly, the synergistic interaction identified in vitro was subsequently confirmed in a murine model of MRSA wound infection, where the formulation eliminated infection and reduced the development of resistance compared to conventional topical antibiotic therapy. Despite generally positive results, bacteriocin efficacy was strongly dependent on the model system and the target pathogen. For instance, nisin demonstrated protective effects in a *Galleria mellonella* infection model against *S. epidermidis*, but failed to improve survival in larvae infected with *E. coli* [90]. Despite promising results, several limitations must be considered when interpreting these studies. Firstly, most in vivo experiments were conducted using murine models which do not fully replicate human skin physiology, immune responses, or microbiota composition. Secondly, the majority of studies focused on monomicrobial infections, whereas clinical wound infections are typically polymicrobial. This limits the ability to predict bacteriocin efficacy in real-world settings. Thirdly, variability in experimental design, including differences in dosing, formulation, and route of administration, complicates direct comparison between studies. Finally, relatively few studies evaluated long-term outcomes, such as recurrence of infection or resistance development, which are critical for assessing therapeutic potential.

In conclusion, current evidence supports bacteriocins as promising candidates for topical treatment of skin and wound infections, especially those involving MRSA and biofilm-forming bacteria. Nevertheless, translation into clinical practice is still limited, as most data have been obtained from in vitro experiments or murine models, and controlled human clinical trials remain scarce.

## 7. Topical Delivery of Bacteriocins: Pharmaceutical Formulations and Translational Perspectives in the Treatment of Skin Infections

Although bacteriocins exhibit potent antimicrobial activity against numerous skin-associated pathogens in vitro, their successful therapeutic application depends largely on overcoming pharmacological and biophysical limitations related to peptide delivery. As ribosomally synthesized peptides, bacteriocins are susceptible to proteolytic degradation, adsorption to biological surfaces, and rapid inactivation in physiological environments, which can markedly reduce their activity when they are applied directly to infected tissues [11,92]. Topical administration is the most appropriate route for dermatological application of antimicrobial peptides. It allows for high local concentrations of a drug at the site of infection, while enables to minimize systemic exposure and toxicity and simultaneously support wound healing processes [92]. However, the stratum corneum constitutes a significant permeability barrier, and wound exudate, proteases, and biofilm matrices may further impair peptide stability and bioavailability. Consequently, recent research has been increasingly focusing on development of dedicated pharmaceutical formulations and delivery systems designed to protect bacteriocins from degradation, prolong their residence time at the infection site, and enhance their penetration into biofilms and infected tissue. A variety of advanced carrier platforms, including hydrogels, electrospun nanofibers, lipid nanoparticles, and bioadhesive scaffolds, were investigated as vehicles for bacteriocin delivery. These systems not only stabilize peptides but also enable controlled and sustained release, maintenance of a moist wound environment, and, in some cases, additional presented wound-healing or immunomodulatory effects. More importantly, several bacteriocin-based formulations have already demonstrated efficacy in animal models of skin infection and wound healing, suggesting their potential for future clinical application in humans. The studies summarized in Table 3 collectively indicate that the clinical translation of bacteriocins will most likely rely on their incorporation into advanced topical delivery platforms rather than their use as conventional antibiotics, positioning these peptides as active components of antimicrobial and biofunctional wound-care systems.

A common feature across the analysed studies is the use of hydrogel-based delivery systems, which provide a moist wound environment and allow for controlled release of the active compound. For example, a hybrid thermosensitive hydrogel incorporating garvicin KS demonstrated prolonged bacteriocin release for up to nine days, with strong antibacterial and antibiofilm activity against *S. aureus* [93]. This extended release profile is particularly advantageous in chronic wound settings, where sustained antimicrobial exposure is required to control biofilm-associated infections. Similarly, a multifunctional hydrogel containing jileicin exhibited not only antimicrobial activity but also additional biofunctional properties, including tissue adhesion, haemostatic effects, and promotion of wound healing [94]. Importantly, this system demonstrated efficacy in a diabetic wound model, suggesting that bacteriocin-based formulations may remain active even in protease-rich environments that typically inactivate peptide therapeutics. Apart from hydrogels, also nanotechnology-based approaches have been explored to enhance bacteriocin stability and efficacy. Incorporation of lacticin 3147 into solid lipid nanoparticles (SLN) significantly improved its physicochemical stability and prolonged antimicrobial activity compared to the free peptide [95]. Encapsulation also provided protection against enzymatic degradation. Electrospun nanofiber dressings represent another promising strategy. Nisin-loaded nanofibers, composed of poly(ethylene oxide) and poly(d,l-lactide), enabled sustained release, significantly reduced bacterial load in vivo, and accelerated wound closure [91]. These systems allow for high local concentrations of bacteriocins at the infection site and simultaneously minimize systemic exposure. Multifunctionality of bacteriocin-based formulations is an important emerging concept. Unlike traditional antibiotics, bacteriocins can be integrated into platforms that simultaneously provide antimicrobial, structural, and regenerative functions. For instance, jileicin-based hydrogels promoted angiogenesis and collagen deposition in addition to reducing bacterial load [94].

Despite promising results, there are still some critical limitations. One of the most important challenges is peptide stability, as bacteriocins such as nisin are prone to proteolytic degradation and may lose activity over time, particularly during storage or in protease-rich wound environments [91]. Furthermore, limited penetration into mature biofilms is a concern, even when advanced delivery systems are used. While sustained release can improve efficacy against early-stage biofilms, complete eradication of established biofilm structures is difficult. Safety and biocompatibility also require further investigation. For example, reduced fibroblast viability was observed in vitro following prolonged exposure to garvicin KS formulations [93], highlighting the need to balance antimicrobial potency with cytotoxicity. A comparative analysis of currently available delivery platforms reveals several common trends. Although hydrogel-, nanoparticle-, and nanofiber-based systems consistently improved bacteriocin stability and local antimicrobial efficacy, no single formulation strategy has yet emerged as clearly superior. Hydrogels offer the additional advantage of supporting wound healing and tissue regeneration, whereas nanoparticle-based systems provide enhanced peptide protection and prolonged release. In contrast, electrospun nanofibers may be particularly attractive for localized delivery directly at the wound surface. Despite these differences, all approaches face similar translational challenges, including limited clinical validation, insufficient long-term safety data, manufacturing complexity, and uncertain scalability.

A lack of comprehensive in vivo and clinical data is another major limitation. While several formulations demonstrated efficacy in animal or in ex vivo models, only few studies have been clinically validated. Moreover, most studies focused on Gram-positive pathogens and hardly evaluated efficacy against Gram-negative bacteria. Despite significant progress in formulation strategies, several challenges that may hinder clinical translation still remain. First of all, many delivery systems are relatively complex, involving multiple components (e.g., liposomes, polymers, bioactive additives), which may complicate large-scale manufacturing and regulatory approval. Moreover, long-term safety data are lacking, particularly those regarding repeated topical application and potential cytotoxic effects on host tissues. Thirdly, most studies focus on Gram-positive pathogens. Gram-negative bacteria are increasingly becoming relevant in chronic wound infections. Yet, efficacy against latter type of bacteria is scarcely evaluated. Finally, stability during storage and maintenance of antimicrobial activity over time are critical concerns, especially for peptide-based therapeutics.

## 8. Discussion

Collective evidence presented in this review highlights bacteriocins as a promising and mechanistically distinct class of antimicrobial agents with potential application in the management of skin and wound infections. Data derived from in vitro susceptibility testing, infection models, and formulation-based studies consistently demonstrate that bacteriocins exhibit potent activity against key Gram-positive pathogens, particularly *S. aureus*, which remains among the most clinically relevant causative agents of wound infections. Across multiple experimental studies, bacteriocins confirmed their strong intrinsic activity against planktonic Gram-positive bacteria. Importantly, several of these peptides also demonstrated antibiofilm activity; however, usually at higher concentrations than those required to inhibit planktonic growth. This distinction underscores a critical challenge in treatment of chronic wounds, where biofilm-associated bacteria exhibit increased tolerance to antimicrobial agents. In contrast, the activity of bacteriocins against Gram-negative pathogens, including *Acinetobacter* spp., *Pseudomonas* spp., and *E. coli*, was markedly less consistent and generally required substantially higher concentrations to achieve inhibitory effects. This limitation is primarily attributed to the presence of the outer membrane, which restricts access of bacteriocins to their cellular targets. Nevertheless, selected studies indicate that certain bacteriocins, particularly those derived from Gram-negative bacteria, may partially overcome this barrier, highlighting thereby an important area for future investigation. In particular, nisin and several bacteriocin-based combinations demonstrated significant reductions in bacterial load and inhibition of biofilm formation in experimental wound infection models, although most currently available evidence concerns infections caused by Gram-positive pathogens. One of the most compelling observations across multiple studies is the enhanced efficacy of bacteriocins when they are used in combination with conventional antibiotics. Synergistic interactions, as demonstrated for nisin with oxacillin or micrococcin P1 with rifampicin, resulted in improved bacterial clearance and, importantly, reduced the risk of resistance development. These findings support the concept that bacteriocins may be most effectively deployed as adjunctive agents rather than standalone therapeutics. The translational potential of bacteriocins is further strengthened by advances in formulation strategies. A wide range of delivery systems, including hydrogels, nanofibers, and lipid-based nanoparticles, have been shown to improve peptide stability, to enable sustained release, and to enhance antimicrobial activity in wound-like environments. Multifunctional platforms, such as bioadhesive or thermosensitive hydrogels, additionally provide structural and regenerative benefits, including improved wound healing, angiogenesis, and tissue integration. These systems position bacteriocins not only as antimicrobial agents but also as integral components of next-generation wound care technologies.

Bacteriocins should also be considered in the broader context of novel antimicrobial strategies aimed at combating antimicrobial resistance and biofilm-associated infections. Other promising approaches include bacteriophage therapy, modified antimicrobial peptides, antimicrobial polymers, and nanomaterial-based antimicrobial systems. These strategies vary in their spectrum of antimicrobial activity, resistance potential, safety, manufacturing requirements, and clinical application. Bacteriophage therapy offers highly selective activity against bacterial pathogens, including multidrug-resistant strains, while potentially preserving a beneficial microbiota. However, its clinical application is limited by the need for phage-pathogen matching, a narrow host range, and the potential for phage resistance [96,97]. Engineered antimicrobial peptides and polymers offer additional potential for combating resistant and biofilm-associated bacteria, but their translation remains hampered by cytotoxicity, physicochemical instability, complex manufacturing, and limited long-term safety data [98]. Nanomaterial-based approaches, including antibacterial photodynamic therapy (aPDT) and antibacterial sonodynamic therapy (aSDT), have also demonstrated broad antimicrobial and antibiofilm activity. Although these approaches may reduce the likelihood of developing conventional resistance, the potential for adaptive tolerance and resistance requires further investigation, and their clinical application may depend on specialized activation systems and delivery platforms [99,100]. Importantly, bacteriocins should not be viewed as direct competitors to these new antimicrobial treatments, but rather as potentially complementary components of multimodal therapies. Their selective activity, antibiofilm potential, and compatibility with advanced topical delivery systems may be particularly beneficial in the local treatment of complex infections. Combining bacteriocins with conventional antibiotics, bacteriophages, or biomaterial-based delivery systems may therefore provide broader and more sustained antimicrobial activity than monotherapy. However, such combinations require experimental validation, as their efficacy may depend on the pathogen, formulation, treatment sequence, and local infection environment.

An important advantage of bacteriocins over conventional broad-spectrum antibiotics may lie in their relatively selective antimicrobial activity. Increasing evidence indicates that preservation of the resident skin microbiota plays a critical role in maintaining barrier integrity, immune homeostasis, and colonization resistance against pathogens [33,39,41]. Broad-spectrum antibiotics can disturb these ecological interactions, promoting dysbiosis and facilitating recolonization by opportunistic microorganisms. In contrast, many bacteriocins exhibit a narrow spectrum of activity directed against specific pathogens while exerting a reduced impact on beneficial commensal bacteria [8,25]. Such a microbiota-sparing approach may be particularly valuable in chronic wound management, where restoration of microbial balance is increasingly recognized as an important component of successful therapy [33,41,42]. Although further studies are required to confirm these effects under clinical conditions, selective targeting of pathogenic microorganisms represents one of the most attractive features of bacteriocin-based therapeutics.

Although bacteriocins are frequently described as exhibiting a lower propensity for resistance development than conventional antibiotics, resistance mechanisms have nevertheless been reported and should not be overlooked. Depending on the bacteriocin class and target organism, resistance may involve modification or loss of specific receptors, alterations in membrane composition and surface charge, changes in transport systems involved in peptide uptake, increased proteolytic degradation, or the action of dedicated immunity proteins produced by bacteria [8,25]. In Gram-positive bacteria, resistance to certain class II bacteriocins has been associated with modifications of the mannose phosphotransferase system (Man-PTS), whereas adaptation to membrane-active peptides may involve alterations in cell envelope architecture and membrane-associated targets [24]. Similarly, Gram-negative bacteria may reduce susceptibility through modifications of outer membrane receptors and uptake pathways required for bacteriocin entry [25,27]. These observations indicate that although resistance appears to develop less frequently than for many conventional antibiotics, it should not be considered negligible. Consequently, long-term surveillance, resistance monitoring, and systematic susceptibility testing will be essential during the future clinical development of bacteriocin-based therapeutics.

Despite the growing body of evidence supporting the antimicrobial efficacy of bacteriocins, their transition into routine clinical practice remains limited. This discrepancy reflects the existence of several translational barriers extending beyond antimicrobial activity itself. Large-scale production of bacteriocins remains technically and economically demanding. Moreover, many bacteriocins exhibit limited stability under physiological conditions due to proteolytic degradation, interactions with biological fluids, and reduced activity in complex infection environments. A successful pathway toward clinical implementation will likely require standardized efficacy testing, optimization of delivery systems, comprehensive pharmacokinetic and safety evaluation, and validation through well-designed clinical trials.

One of the principal challenges is the limited stability of bacteriocins in the skin environment. The surface of the skin is characterized by an acidic pH, high salt concentration, lipid-rich secretions, and the presence of host and bacterial proteases. These conditions can lead to rapid degradation or inactivation of peptide antimicrobials, particularly in chronic wounds, where proteolytic activity is markedly elevated [92]. Consequently, direct application of purified bacteriocins in solution often results in a short duration of activity and insufficient local concentrations. Another key limitation is retention at the site of infection. Unlike systemic antibiotics, bacteriocins are most suitable for topical administration; however, effective therapy requires prolonged contact with the infected tissue. Wound exudate, mechanical removal, and dilution by tissue fluids can rapidly reduce local peptide concentration below bactericidal levels [95]. This problem is especially relevant in chronic wounds and burn injuries, where continuous fluid secretion prevents stable drug exposure. Application of bacteriocins as antibiotic adjuvants is another promising solution. Several studies have demonstrated synergistic interactions between bacteriocins and conventional antibiotics, reducing antibiotic dosage requirements and restoring susceptibility in resistant strains [88]. Bacteriocins can enter clinical practice as combination therapies aimed at combating antimicrobial resistance rather than replace antibiotics entirely. Bacteriocins are biological molecules requiring scalable production, purification, and assurance of stability. Variability between batches, potential immunogenicity, and the need for standardized pharmacokinetic evaluation complicate approval pathways [8,11].

Although bacteriocins have demonstrated promising antimicrobial properties, several aspects relevant to clinical translation remain insufficiently investigated. In particular, pharmacokinetic data are scarce and long-term toxicity and immunogenicity profiles remain poorly characterized. Furthermore, only limited clinical evidence is currently available, highlighting the need for well-designed clinical studies capable of evaluating efficacy and safety under clinically relevant conditions. Despite these limitations, recent evidence suggests that the mechanistic diversity and ecological adaptability of bacteriocins continue to support their development as promising antimicrobial agents with considerable translational potential [101]. Manufacturing scalability and regulatory approval pathways will also play important roles in determining the future clinical implementation of bacteriocin-based therapeutics. Therefore, interpretation of currently available data should be approached with caution due to several limitations of the existing literature. Considerable heterogeneity exists among studies with respect to bacteriocin purification methods, susceptibility testing protocols, biofilm models, bacterial strains, outcome measures, and experimental conditions. Such variability complicates direct comparisons between studies and limits the establishment of standardized efficacy benchmarks [1,6]. Furthermore, the majority of investigations have been performed using in vitro assays or animal models, while clinical evidence remains extremely limited. Most studies have focused on monomicrobial infections and Gram-positive pathogens, despite the polymicrobial nature of many chronic wound infections and the increasing clinical importance of Gram-negative bacteria in wound microbiology [1,64]. Biofilm-associated infections themselves are highly heterogeneous and may differ substantially between experimental systems and clinical wounds, which further complicates translation of laboratory findings into clinical practice [6,66].

Consequently, future research should prioritize standardized methodologies, clinically relevant polymicrobial models, and well-designed clinical studies to facilitate objective assessment of the therapeutic potential of bacteriocins in skin and wound infections. Overall, the available evidence suggests that bacteriocins are unlikely to emerge initially as systemic antibiotics. Instead, their most realistic near-term clinical application lies in localized therapies, particularly chronic wounds, burn infections, acne-associated infections, and MRSA decolonization, where high local concentrations can be achieved and systemic toxicity minimized.

## 9. Conclusions

In conclusion, this review demonstrates that bacteriocins represent a promising class of antimicrobial agents with potent activity against Gram-positive wound pathogens and emerging potential in the management of biofilm-associated infections. Their successful clinical application will likely depend on the integration of antimicrobial activity with advanced delivery systems capable of improving peptide stability, retention, and local bioavailability within wound environments. However, several key knowledge gaps remain. Future research should prioritize (i) evaluation of bacteriocins in mature and polymicrobial biofilm models that better reflect chronic wound infections, (ii) standardization of antimicrobial and antibiofilm testing methodologies to facilitate comparison between studies, (iii) optimization of delivery systems ensuring sustained local activity, (iv) comprehensive assessment of toxicity, immunogenicity, and pharmacokinetic properties, and (v) well-designed clinical studies validating efficacy and safety in patients. Addressing these challenges will be essential for translating promising experimental findings into clinically applicable bacteriocin-based therapies for skin and wound infections.

## Figures and Tables

**Table 1:** Antimicrobial activity of bacteriocins against bacterial species relevant to skin and wound infections.

Bacteriocin	Producer	Target Pathogen(s)	Antibacterial Activity	Biofilm Activity	Ref.
Nisin A	*Lactococcus lactis*	*S. aureus* *S. epidermidis*	MIC 4 µg/mL (MRSA 8 µg/mL)	MBIC = 4 µg/mL MBBC = 4 to 256 µg/mL	[13]
Nisin A	*L. lactis*	*S. aureus*	MIC 2–256 µg/mL (strain-dependent)	Reduces biofilm formation after 24 and 48 h	[14]
		*P. aeruginosa*	MIC 64 µg/mL (strain-dependent)		
Nisin A	*L. lactis*	*S. aureus*	MIC 4–16 µg/mL	Not reported	[69]
Nisin A	*L. lactis*	*Acinetobacter* spp. *Pseudomonas* spp. *Escherichia* spp. *Klebsiella* spp. *Proteus* spp. *Serratia* spp.	MIC 1.65–26.40 µg/mL MIC 6.60–423 µg/mL MIC 26.40–423 µg/mL MIC 105.70–432 µg/mL MIC 105.70 µg/mL MIC 13.20–423 µg/mL	Not reported	[70]
Enterocin AS-48	*Enterococcus faecalis*	*S. aureus*	MIC 3 to 16 mg/L	Biofilm reduction	[71]
Capidermicin	*S. capitis*	*S. aureus*	MIC 3.1 to 10 µg/mL	Not reported	[72]
Lacticin 3147 and Nisin	*L. lactis*	*S. aureus*	MIC _lacticin 3147_ 1.9–61.8 mg/L MIC_nisin_ 0.5–8.3 mg/L	Not reported	[73]
		*Enterococcus* spp.	MIC_lacticin 3147_ 1.9–7.7 mg/L MICnisin 2–8.3 mg/L		
Bacteriocin from *Bacillus subtilis*	*B. subtilis*	*S. aureus*	MIC 32 µg/mL	Not reported	[74]
Plantaricin GZ1-27	*L. plantarum*	*S. aureus*	MIC 32 µg/mL	Not reported	[75]
Plantaricin ZFM9	*L. plantarum*	*S. aureus*	MIC 1.5 µM	Not reported	[76]
Bacteriocin from *L. plantarum*	*L. plantarum*	*S. aureus*	MIC 15–30 µg/mL	Not reported	[77]
Epidermicin NI01	*S. epidermidis*	*S. epidermidis* *S. aureus* *E. faecalis*	MIC 0.0625–4 µg/mL MIC 1–2 µg/mL MIC 0.5–2 µg/mL	Not reported	[78]
Gallidermin	*Staphylococcus* *gallinarum*	*S. aureus* *S. epidermidis*	MIC 6.25 µg/mL MIC 1.56–12.5 µg/mL	Not reported	[79]
Bacteriocin from *L. plantarum* PTCC 1745	*L. plantarum*	*Acinetobacter baumanii*	MIC 30–120 µg/mL MBC 60–120 µg/mL	59% reduction in biofilm formation	[80]
Bacteriocin HA2-5	*Bacillus haynesii* HA2	*C* *. acnes* *S. aureus*	MIC: 8 µg/mL MIC 32 µg/mL	Not reported	[81]
Flexusin A	*Bacillus flexus* R29-2	*S. aureus* *E. faecalis* *E. faecium*	MIC: 1 µg/mL MIC: 16 µg/mL MIC: 32 µg/mL	Not reported	[82]
Bacteriocin PA166	*Pseudomonas* spp.	*Pasteurella multocida*	MIC 1–2 µg/mL	Not reported	[83]
Bacteriocin from *P. aeruginosa*	*P. aeruginosa*	*Staphylococcus* spp. *E. coli* *Enterobacter* spp. *Klebsiella* spp.	MIC 6.15 μM MIC 3.6 μM MIC 12.5 μM MIC 10.2 μM	Not reported	[84]

The table summarized antimicrobial activity of bacteriocins against bacterial species relevant to skin and wound infections, including planktonic and biofilm-associated models. MIC—minimum inhibitory concentration; MBIC—minimum biofilm inhibitory concentrations; MBBC—minimum biofilm bactericidal concentrations.

**Table 2:** Experimental evidence for the use of bacteriocins in skin and wound infection models.

Bacteriocin	Producer	Target Pathogen(s)	Model System	Infection Type/Outcome	Key Findings	Translational Relevance	Ref.
Nisin A + oxacillin	*L. lactis*	*S. aureus* (MRSA)	In vitro + murine skin infection model	MRSA skin infection	Synergistic antibacterial effect; inhibition of biofilm formation; significant reduction in the amount of bacteria in infected skin.	Supports combination therapy as a strategy to enhance MRSA eradication and reduce biofilm-associated persistence in wound infections.	[87]
Bacteriocin XJS01	*L. salivarius* XJS01	*S. aureus*	In vitro + murine infection model	Biofilm-associated *S. aureus* wound infections	Reduction the number of bacteria in the biofilm formed on the wound surface.	Suggests potential use as a topical antibiofilm agent for chronic wound infections.	[12]
Micrococcin P1 + rifampicin	*Macrococcus caseolyticus*	*S. aureus* (MRSA)	In vitro + murine infection model	MRSA wound infection	Combination therapy efficiently eradicated MRSA and prevented relapse.	Supports adjunctive therapy to improve efficacy and reduce recurrence.	[88]
Garvicin KS + micrococcin P1 + penicillin G	*Lactococcus garvieae*/*M. caseolyticus*	*S. aureus* (MRSA)	In vitro + murine infection model	MRSA wound infection	Three-component formulation eradicated MRSA from wounds and reduced resistance development compared to topical antibiotic.	Demonstrates the potential of multi-component antimicrobial therapies for difficult-to-treat MRSA wound infections and resistance prevention.	[89]
Nisin A	*L. lactis*	*S. epidermidis* and *E. coli*	In vitro, ex vivo porcine wound healing model, model *Galleria mellonella*	*S. epidermidis* and *E. coli* infection	Nisin A treatment, did not confer protection to the larvae against the *E. coli* infection but increased the survival of the larvae infected with *S. epidermidis.*	Indicates pathogen-dependent efficacy and highlights the need for pathogen-specific therapeutic applications.	[90]
Nisin A	*L. lactis*	*S. aureus* (MRSA)	Murine infected wound model	MRSA wound infection	Local delivery of nisin significantly reduced bacterial counts in infected wounds, prevented progression of infection.	Provides evidence supporting local delivery of bacteriocins for MRSA wound management.	[91]

**Table 3:** Formulation strategies and delivery systems for bacteriocins in skin infection therapy.

Bacteriocin	Formulation/Delivery System	Target Skin Application	Key Advantages	Limitations/Challenges	Ref.
Nisin A	Nanofiber wound dressing (nisin + poly(ethylene oxide) and poly(d,l-lactide) blend nanofibers	*S. aureus* wound infection	Sustained peptide release, high local concentration, strong bacterial reduction in vivo, promotes wound closure, protects peptide from proteolysis.	Limited penetration of peptides into bacteria in biofilm, degradation of peptides during long-term storage.	[91]
Garvicin KS peptides	Hybrid thermosensitive hydrogel based on Pluronic F127 incorporating EDTA-loaded liposomes and glutathione	Infected chronic wounds; MRSA infected wounds; biofilm-associated skin infections	Sustained bacteriocin release (up to ~9 days); strong antibacterial and antibiofilm activity against *S. aureus.*	Reduced fibroblast viability observed in vitro at prolonged exposure; limited activity against Gram-negative bacteria.	[93]
Jileicin (JC)	Bioadhesive multifunctional hydrogel composed of hyaluronic acid and gelatin	Infected diabetic wounds; MRSA-infected skin wounds (murine model)	High tissue adhesion and hemostatic properties; broad activity against Gram-positive pathogens; immunomodulatory effects and accelerated wound healing in vivo	Complex formulation; regulatory classification (device + drug); storage stability.	[94]
Lacticin 3147	Solid lipid nanoparticle (SLN)-loaded hydrogel for topical application	*S. aureus*-infected skin; wound infection model (ex vivo porcine skin)	Prolonged antimicrobial activity compared to free bacteriocin; improved physicochemical stability; effective against Gram-positive wound pathogens, improved activity against biofilm bacteria.	No in vivo wound-healing model.	[95]

Delivery systems such as hydrogels, wound dressings, nanofibers, nanoparticles, and combination therapies, along with their reported advantages and developed experimental models were highlighted in the table.

## Data Availability

No new data were created or analyzed in this study. Data sharing is not applicable.
